# Notes on Two Rare Species of Brachyuran Crabs (Families Matutidae and Parthenopidae) From Indonesian Waters with New Distribution Records

**DOI:** 10.21315/tlsr2023.34.3.10

**Published:** 2023-09-30

**Authors:** Nisfa Hanim, Ali Suman, Duranta Diandria Kembaren, Dyah Perwitasariv, Yusli Wardiatno, Achmad Farajallah

**Affiliations:** 1 Department of Biology, Faculty of Mathematics and Natural Sciences, IPB University, Kampus IPB Dramaga, Bogor, West Java, Indonesia; 2 Ministry of Marine Affairs and Fisheries, Research Institute for Marine Fisheries, 14430 Jakarta, Indonesia; 3 National Research and Innovation Agency (BRIN), Research Centre for Fishery, 16912 Bogor, Indonesia; 4 Department of Aquatic Resources Management, Faculty of Fisheries and Marine Sciences, IPB University, Kampus IPB Dramaga, Bogor, West Java, Indonesia

**Keywords:** Biodiversity, Dispersal, Elbow Crab, Marine, Systematics

## Abstract

Several species of brachyuran crabs in Indonesian waters have not been reported since almost 100 years ago. This research reports a brachyuran crab that is rarely found and one new record in Indonesian waters. This study was conducted in the waters of southern Aru Island and the Malacca Strait using trawls during a cruise held by the Research Institute for Marine Fisheries, Ministry of Marine Affairs and Fisheries, Indonesia.. Our findings were *Izanami reticulata*, a new record (family Matutidae) from southern Aru Island, and *Cryptopadia fornicata* (family Parthenopidae) from the Malacca Strait. The two locations are close to where the species were found in previous studies: the Arafura Sea, which is adjacent to the Aru Islands, and the Malacca Strait, which is adjacent to Borneo. It is suspected that the presence of these two species in Indonesia is due to their distribution through sea currents during the pelagic larval stage. This article also provides the specific habitat for both species in Indonesia, which was previously unknown. In addition, this article contributes to strengthening Indonesia as a mega-biodiversity country with an initial compilation of a database of Brachyura in its waters.

**Highlights**

- *Izanami reticulata* P. Müller and Galil, 1998 as a new record in Indonesia.
- Strengthening the presence of *Cryptopodia fornicata* J. C. Fabricius, 1781 in Indonesia.
- The distribution of species family Matutidae in Indonesia.

## INTRODUCTION

The infraorder Brachyura is part of the order Decapoda, which has more than 6,000 species inhabiting land and oceans (Ng *et al*. 2008). Studies on Brachyura have been conducted intensely in almost all regions of the world. In Indonesia, several expeditions have been carried out to study marine biotas (Table 1). However, information regarding species of the infraorder Brachyura in Indonesia is still relatively scarce (Moosa & Hutomo 2005), several groups of Brachyura have never been reported again, and similarly for the genera of *Izanami* Galil & P. F. Clark, 1994 and *Cryptopodia* H. Milne Edwards, 1834.

*zanami* has short lateral spines that separate them from *Matuta* Weber 1795, and this genus has only two species, namely *Izanami reticulata* P. Müller & Galil 1998 and *Izanami curtispina* T. Sakai 1961 (Galil & Clark 1994). Both species have been found in waters adjacent to Indonesia (Australia [Arafura Sea], Philippines, Japan, China Sea, Madagascar and New Caledonia). Unfortunately, there have been no reports about their occurrence within Indonesia’s marine territory.

*Cryptopodia* (family Parthenopidae) has a restricted distribution in the Indo-West Pacific region, at depths of 10 m–30 m. It consists of 12 species (Ng & Chiong 1998), of which only four have been reported in Indonesian waters, namely *C. angulata* H. Milne Edwards & Lucas, 1841 (the specific location not mentioned; Yang 1979), *C. collifer* Flipse, 1930 (Siboga Expedition; Flipse 1930), *C. laevimana* Miers, 1879 (Borneo; Miers 1879; Ng & Chiong 1994), and *C. fornicata* J. C. Fabricius, 1781 (Borneo; Miers 1884; Irian Jaya; Flipse 1930). Most species were reported in Indonesia more than one century ago. Thus, research conducted in Indonesia on this genus is scarce.

## MATERIALS AND METHODS

Sampling collection was conducted in July 2015 in the Malacca Strait and in November 2018 in the waters of southern Aru Island during cruise research instigated by the Research Institute for Marine Fisheries, Ministry of Marine Affairs and Fisheries, Indonesia. Four specimens were collected from each location using trawl gear in the morning (8.00–11.00 a.m. local time, UTC+7 in Malacca Strait and UTC+9 in southern Aru Island). Samples from each location were preserved in formalin and ethanol 96%, respectively. All four specimens were deposited in the Biosystematics and Animal Ecology Laboratory, Department of Biology, IPB University, with specimen numbers A1 (male), A2 (female) (southern Aru Island), and K40 (male) and K66 (female) (Malacca Strait). Specimens were photographed using a Nikon camera (COOLPIX B700, Nikon, Japan) and then measured using a calliper. Identification at the species level followed Galil and Clark (1994) and Ng and Chiong (1998). Information about the measure was available, Carapace Width (CW) and Carapace Length (CL). CW was measured from both the tip of the posterolateral tooth, and CL was measured from the rostrum until the posterior border of carapace using caliper.

## RESULTS

### *Izanami reticulata* P. Müller & Galil, 1998, Southern Aru Island

Family Matutidae De Haan, 1835 Class Malacostraca Latreille, 1802 Order Decapoda Latreille, 1802 Infraorder Brachyura Linnaeus, 1758

Superfamily Calappoidea De Haan, 1833 Family Matutidae De Haan, 1835 Genus *Izanami* Galil & P. F. Clark, 1994

**Figure 1:**
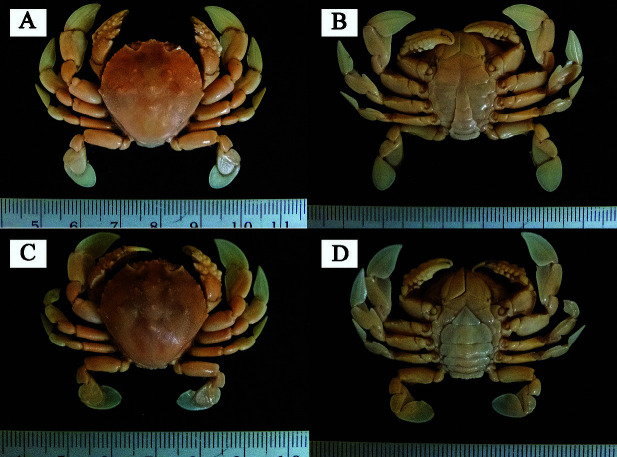
(A) *Izanami reticulata* from southern Aru Island (dorsal view, male); (B) *I. reticulata* (ventral view, male); (C) *I. reticulata* (dorsal view, female); (D) *I. reticulata* (ventral view, female).

*I. reticulata* P. Müller & Galil, 1998, new record (Fig. 1)

*Matuta inermis* Miers, 1884: pp. 256–257, Fig. C, Plate XXVI (type locality Albany Island, Torres Strait).

*Izanami inermis* (Miers, 1884): Galil and Clark, 1994: pp. 28–31, Figs. 5c–5d, Plate 10a–10b

### Examined Material

Indonesia. One male, CW = 25.5 mm, CL = 26.3 mm and one female, CW = 24.3 mm, CL = 24.4 mm; southern Aru Island waters; 06°59.363′N, 134°3.693′E; 27 m depth; sandy substrate; 12 November 2018; collector: D. D. Kembaren.

### Diagnosis

Carapace circular. Anterolateral surface carapace granulated; frontal median lobe bifid. Surface carapace rough, with six tubercles. Tubercle in cardiac region longest. Posterolateral teeth very short.

### Description

Carapace circular, measures of its length and width similar. Surface carapace granulated, particularly in the anterolateral region, with six tubercles present. Tubercle in cardiac region longest (1.45 mm in female and 1.36 mm in male) and placed between two other smaller tubercles on right and left sides. Carapace smooth posteriorly. Anterolateral margin of carapace has three small teeth, not including inner orbital lobes, tuberculated. Posterolateral teeth rudimentary (very short). Frontal region carapace has three lobes. median lobe (rostrum) bifid, two lateral lobes arranged horizontally (Figs. 1A and 1C).

Measurements of both chelipeds similar, 60 mm. Merus short, slightly slender, smooth. Carpus short, swollen, tuberculated. A tuberculate ridge in upper margin of carpus and a sharp tooth at an angle. Palm slightly longer. Its upper margin tridentate, and its size diminishing distally. Merus and carpus of walking legs (I–III) granulated anteriorly.

In abdomen of male, telson slightly longer than its width (Fig. 1B), whereas in female, it an isosceles triangle in shape (Fig. 1D). Penultimate segment widest in both male and female (Figs. 1B and 1D).

### Distribution

Southern Aru Island (this study), Australia (Arafura Sea), Philippines and New Caledonia (Galil & Clark 1994).

### Remarks

*I. reticulata* was identified for the first time by Miers (1884) with its type locality in North Australia (Albany Island, Torres Strait), and the scientific name was *M. inermis*. They were also reported in adjacent waters (Thursday Island and Prince of Wales Island, Arafura Sea) by Miers (1884). This species was found in southern Aru Island in Indonesian waters, which is located near the Arafura Sea.

### *Cryptopodia fornicate* J. C. Fabricius, 1781, Malacca Strait

Family Parthenopidae MacLeay, 1838

Class Malacostraca Latreille, 1802

Ordo Decapoda Latreille, 1802

Infraorder Brachyura Linnaeus, 1758

Superfamily Parthenopoidea MacLeay, 1838

Family Parthenopidae MacLeay, 1838

Genus *Cryptopodia* H. Milne Edwards, 1834

**Figure 2:**
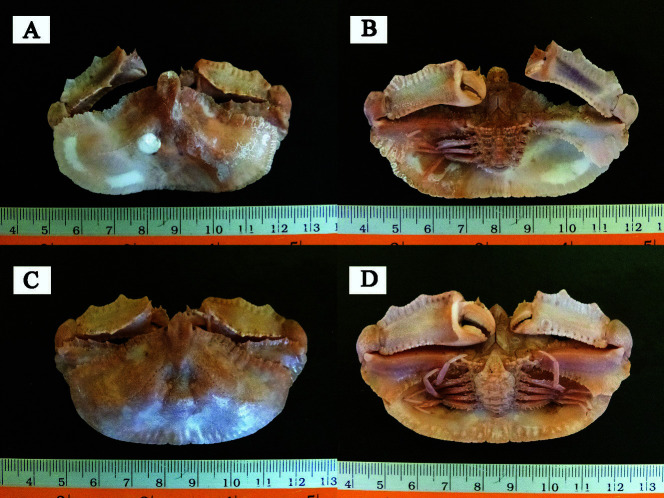
(A) *Cryptopodia fornicate* from Malacca Strait (dorsal view, male); (B) *C. fornicata* (ventral view, male); (C) *C. fornicata* (dorsal view, female); (D) *C. fornicata* (ventral view, female).

*C. fornicata* J. C. Fabricius, 1781 (Fig. 2)

*Cancer fornicata* Fabricius, 1781: p. 502

*Cryptopodia pentagona* Flipse, 1930: pp. 67–68, Fig. 42

### Examined Material

Indonesia. One male, CW = 66.6 mm, CL = 37.8 mm, and one female, CW = 66.8 mm, CL = 41.0 mm; Malacca Strait; 02°54.444′N, 100°47.094′E; ca. 25.7–31.9 m depth; sandy substrate; 1 July 2015; collector: D. D. Kembaren.

### Diagnosis

Carapace broader than long. Lateral sides of carapace have wide expansions. The branchial, cardiac, and gastric regions are strongly inflated, and the lateroventral carapace depression is deep.

### Description

Carapace broader than long, >1.5 times its length, and pentagonal. Lateral sides of carapace have wide expansions concealing all ambulatory legs. Anterolateral margin carapace denticulated (Figs. 2A and 2C). Posterolateral margin crenulated and can be seen more clearly in male (Fig. 2B). Posterior margin carapace nearly straight (Figs. 2C and 2D), but slightly concave in male specimen (Figs. 2A and 2B) and crenulated. Branchial, cardiac, and gastric regions elevated and form a shallow triangular indentation in centre of carapace (Figs. 2A and 2C). Mesobranchial and metabranchial ridges granulated. Rostrum broader than long and triangular in female (Figs. 2C and 2D), whereas it more rounded in male (Figs. 2A and 2B). Surface of ventral carapace smooth, with a deep lateroventral carapace depression (Figs. 2B and 2D).

Right cheliped slightly larger than left. Anterior facet of merus consists of three prominent teeth of equal size that denticulated. Distal part of posterior facet of merus has a wing-like expansion that denticulated. Carpus small. Anterior margin of dorsal facet of palm has a slight expansion and denticulated. Posterior margin of dorsal facet of palm has five prominent teeth (Figs. 2A–2D).

Ambulatory legs slender; first pair longest, and next diminish in size. Merus has setae (Figs. 2B and 2D).

Abdomen granulated in both male and female, and telson triangular (Figs. 2B and 2D).

### Distribution

Malacca Strait (this study), Indonesia (Borneo; Miers 1884; Irian Jaya; Flipse 1930); Singapore, Philippines, Thailand, Japan, Malaysia and China (Ng & Chiong 1998).

### Remarks

The results confirm the occurrence of *C. fornicata* i n Indonesian w aters. *C. fornicata* was confirmed as the correct name for *C. queenslandi* Rathbun, 1918 and *C. patula* Chiong & P. K. L. Ng, 1998 (Ng & Chiong 1998). It was first reported in Borneo, Indonesia as *C. fornicata* by Miers (1884), but Ng and Chiong (1998) stated (when they reexamined the specimens) that the specimen from Borneo could be *C. fornicata* because there were no reports about its related species (*C. queenslandi* and *C. patula*) from there. Ng and Chiong (1998) also confirmed that *C. pentagona* Flipse, 1930 from Irian Jaya reported by Flipse (1930) was a juvenile of *C. fornicata*.

## DISCUSSION

In the family Matutidae, initially, the genus *Izanami* was the genus *Matuta*, but after a revision by Galil and Clark (1994), *Izanami* and two other genera (*Ashtoret* Galil & Clark, 1994 and *Mebeli* Galil & Clark, 1994) emerged as new genera. Of the three genera of the family Matutidae found in Indonesian waters (*Matuta*, *Ashtoret* and *Izanami*), we can see the distribution pattern of these family groups. For the genus *Matuta*, of the four species found in Indonesia (*M. planipes* Fabricius, 1798, *M. victor* Weber, 1795, *M. circulifera* Miers, 1880, and *M. purnama* J. C. Y. Lai & Galil, 2007), most are distributed in the coastal areas of western Indonesia (Miers 1880; Galil & Clark 1994; Hanim *et al*. 2021), except for *M. planipes* and *M. victor*, which are also found in eastern Indonesia (Galil & Clark 1994). *M. purnama* is found only in coastal areas facing the Indian Ocean (ocean coast) (Lai & Galil 2007; Hanim *et al*. 2021). Although the genus *Matuta* is generally inhabitant of tropical sandy shores (Lai & Galil 2007), *M. victor* is also found in the subtropical region of the Mediterranean Sea (invasive species) (Galil & Mendelson 2013; Innocenti *et al*. 2017). In contrast, the genus *Asthoret* is primarily distributed in eastern Indonesia (Galil & Clark 1994), and the genus *Izanami* is also found in eastern Indonesia at 27 m (this study).

Indonesian waters are connected, resulting in Indonesian throughflows (Hasanudin 1998). This situation could result in a wide distribution of marine biotas with a long planktonic larval phase, one of which is in the brachyuran crab (Fisher 2006). One factor influencing these larvae to settle in water is the availability of nursery habitats, such as seagrass meadows (Epifanio & Cohen 2016), mangroves, and corals (Lefcheck *et al*. 2019). The diversity of these habitats is high in Indonesia.

## CONCLUSION

This study recorded a new distribution of *I. reticulata* in Indonesian waters (as the first report), namely, in the waters of southern Aru Island. This research also confirms the presence of *C. fornicata* in Indonesia (found in the Malacca Strait), where in previous reports there were descriptions of juvenile individuals.

## Figures and Tables

**Table 1:** The series of expeditions to study marine biotas in Indonesia.

No.	Year	Expedition (References)	The researcher of infraorder Brachyura
1	17th century	*Rumphius Biohistorical Expedition to Ambon* (Strack 1993)	Strack (1993); Serene *et al*. (1976)
2	1873–1876	*The Voyage of H.M.S. Challenger* (Miers 1885)	Miers (1885)
3	1899–1900	Siboga Expedition (Weber 1902)	Leene (1938); Leene & Buitendijk (1952) Holthuis & Manning (1990); Galil (2001)
4	1922	The Danish Expedition to the Kei Island (Stephenson 1972)	Stephenson (1972)
5	1929–1930	Snellius Expedition (Wijsman-Best 1974)	Buitendijk (1941); Leene (1940)
6	1950–1952	Galathea Expedition	Stephenson (1972); Griffin & Tranter (1986)
7	1965	Baruna Expedition (Nontji 2017)	Romimohtarto (1967)
8	1990	Rumphius II Expedition	Spiridonov (1999)
9	2007	Widya Nusantara Expedition, routine every year (Wahyudi *et al*. 2016)	
10	2018	South Java Deep Sea Biodiversity Expedition (SJADES 2018)	Mendoza & Nugroho (2021)
